# Wiki-Pi: A Web-Server of Annotated Human Protein-Protein Interactions to Aid in Discovery of Protein Function

**DOI:** 10.1371/journal.pone.0049029

**Published:** 2012-11-28

**Authors:** Naoki Orii, Madhavi K. Ganapathiraju

**Affiliations:** 1 Department of Biomedical Informatics, University of Pittsburgh, Pittsburgh, Pennsylvania, United States of America; 2 Language Technologies Institute, Carnegie Mellon University, Pittsburgh, Pennsylvania, United States of America; Semmelweis University, Hungary

## Abstract

Protein-protein interactions (PPIs) are the basis of biological functions. Knowledge of the interactions of a protein can help understand its molecular function and its association with different biological processes and pathways. Several publicly available databases provide comprehensive information about individual proteins, such as their sequence, structure, and function. There also exist databases that are built exclusively to provide PPIs by curating them from published literature. The information provided in these web resources is protein-centric, and not PPI-centric. The PPIs are typically provided as lists of interactions of a given gene with links to interacting partners; they do not present a comprehensive view of the nature of both the proteins involved in the interactions. A web database that allows search and retrieval based on biomedical characteristics of PPIs is lacking, and is needed. We present Wiki-Pi (read Wiki-π), a web-based interface to a database of human PPIs, which allows users to retrieve interactions by their biomedical attributes such as their association to diseases, pathways, drugs and biological functions. Each retrieved PPI is shown with annotations of both of the participant proteins side-by-side, creating a basis to hypothesize the biological function facilitated by the interaction. Conceptually, it is a search engine for PPIs analogous to PubMed for scientific literature. Its usefulness in generating novel scientific hypotheses is demonstrated through the study of IGSF21, a little-known gene that was recently identified to be associated with diabetic retinopathy. Using Wiki-Pi, we infer that its association to diabetic retinopathy may be mediated through its interactions with the genes HSPB1, KRAS, TMSB4X and DGKD, and that it may be involved in cellular response to external stimuli, cytoskeletal organization and regulation of molecular activity. The website also provides a wiki-like capability allowing users to describe or discuss an interaction. Wiki-Pi is available publicly and freely at http://severus.dbmi.pitt.edu/wiki-pi/.

## Introduction

Annotations of proteins such as their sequence, structure, interactions and functions, or their association to diseases and drugs, are provided by a number of web-based databases such as Uniprot [1], HPRD [2], Gene Cards [3], Gene Ontology [4], KEGG [5], PDB [6], OMIM [7] and REACTOME [8]. Some databases such as BioGRID [9], STRING [10], DIP [11], MINT [12], InnateDB [13], and IntAct [14] are designed exclusively to provide information about protein-protein interactions (PPIs). These PPI databases provide a valuable resource by curating experimentally known interactions, and have become the gold-standard data sources for a number of bioinformatic studies such as prediction of protein-protein interactions and protein functions, gene prioritizations and other systems biology studies. The contribution of most of these websites is the presentation of datasets that are painstakingly compiled by curators from literature. Conversely, a *crowdsourcing* model for curating protein annotations was explored by WikiGenes [15]. Similar to Wikipedia, users can collaboratively create, edit and update articles on the site. Thus, instead of a small group of creators, researchers around the globe are able to contribute to that knowledge base. However, all of these web-based data resources provide a gene-centric view of interactions. That is, the “central players” of these databases are genes and not the interactions. In most of these web resources, interactions are merely provided as lists with respect to a specific protein, and any information about the interactions, if provided, is about the type of interaction or the experimental method or publication that reports the said interaction. Although the information that an interaction exists between two proteins is useful by itself, it may be insufficient from a biomedical researcher's perspective. Biomedical researchers often have one or a few proteins that they study in detail, and exploring the interactions of these proteins requires rich annotations about the interacting partners in order to identify an interaction that is relevant to their research – namely, an interaction that would potentially lead to further experiments in their own lab.

Currently there is no search engine that allows retrieval of PPIs by their biomedical associations. Existing databases primarily allow a user to search for interactions by gene symbol or other widely used identifiers, be it protein/gene name, Entrez gene identifier, or Ensembl identifier. However, biologists specializing in the study of a certain disease or pathway may be interested in retrieving interactions associated with that disease or pathway, and not by a single gene. For example, a researcher studying diabetes is not able to retrieve PPIs associated with diabetes using any of the existing PPI databases (although specialized databases may exist occasionally for a few well-studied diseases). InnateDB and IntAct provide search functionality, and users can search for PPIs by experimental details but not by specifying biomedical attributes of the proteins.

PPIs can contribute to the discovery of a gene's biological function. An example where PPIs have contributed to the discovery of gene function is *Disrupted in Schizophrenia 1* (DISC1), a novel protein discovered in 2000 with no known homolog in human. DISC1 was identified to be associated with schizophrenia; although it had well characterized protein domains such as coiled-coil domains, leucine-zipper domains, and nuclear localization and export signals, nothing was inferred about its function [16], [17]. To understand the function of DISC1, PPIs were determined using yeast 2-hybrid technology [18], [19]. Availability of this ‘DISC1 interactome’ has led to a large number of studies that concluded the association of DISC1 to cAMP signaling, axon elongation and neuronal migration, and accelerated the research pertaining to schizophrenia in general and DISC1 in particular [20]. Therefore, it is useful to have a web resource of PPIs that displays not only the symbols of interacting partners but also comprehensive information on what the interacting partners of a gene can tell about the gene itself.

We developed a web resource, Wiki-Pi, which addresses the above issues. It provides an effective means to search and retrieve interactions of interest, and displays the retrieved interactions with annotations of their biomedical associations so as to enable further discoveries. The search for interactions can be carried out by specifying biological and disease-relevant annotations of genes. Wiki-Pi provides the seed information necessary for gene function discoveries, by readily presenting the annotations of the gene at hand as well as those of its interacting partners. Further, Wiki-Pi facilitates *knowledge-creation via crowdsourcing*. It allows users to discuss or describe their hypothesis, or other known facts that are not part of existing database, in the wiki portion of each interaction. The website is freely available at http://severus.dbmi.pitt.edu/wiki-pi and is viewable in all major browsers including those on smartphones and e-readers.

## Data and Functionality

**Figure 1:**
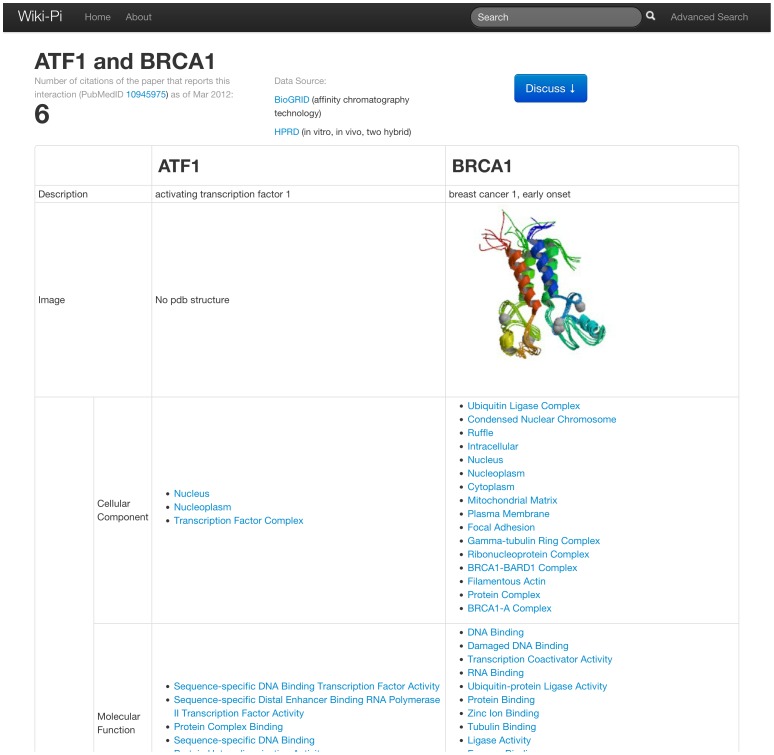
PPI page. A PPI page showing annotations about the proteins and about the interaction. URL: http://severus.dbmi.pitt.edu/wiki-pi/index.php/pair/view/466/672.

**Figure 2:**
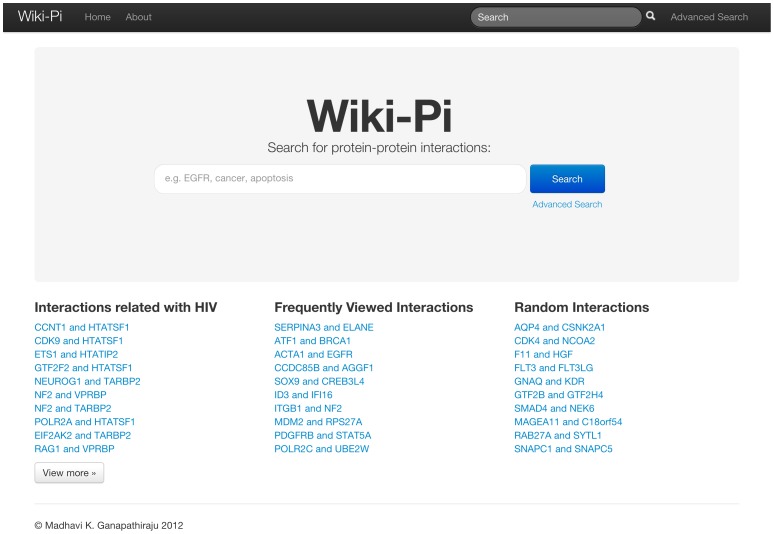
Website homepage. The homepage gives a search box, and also shows a shortlist of interactions some of which are populated randomly from the database while others are those that are most-frequently searched on Wiki-Pi. URL: http://severus.dbmi.pitt.edu/wiki-pi/.

Wiki-Pi is a web resource whose focus is on telling the story of each interaction in the human interactome. Only binary biophysical interactions are presented. Each interaction can be viewed on its own webpage (Figure 1). The mechanism to reach individualized PPI pages is via the search functionality provided on the homepage (Figure 2) or via a search box provided conveniently at the top of any page.

### Data Sources

Binary biophysical interactions of the human interactome have been collected from HPRD and BioGRID. Currently, Wiki-Pi contains 48,419 unique interactions among 10,492 proteins. Data sources for annotations are given in Table 1. Excluding HPRD, all of the data from the databases is automatically updated monthly. Only data from HPRD is updated manually (we note that HPRD has not updated its database since April 13, 2010). We rely on these databases for curated PPIs, and do not curate them from other resources ourselves. The database of interactions and other annotations are loaded into MySQL.

**Table 1:** Data sources.

Data type	Data source	Link
Interactions	HPRD	http://www.hprd.org/ (BINARY_PROTEIN_PROTEIN_INTERACTIONS.txt)
Interactions	BioGRID	http://thebiogrid.org/downloads/archives/Latest%20Release/BIOGRID-ALL-LATEST.mitab.zip
Entrez ID	NCBI Gene	ftp://ftp.ncbi.nih.gov/gene/DATA/gene_info.gz
GO annotations	NCBI Gene2go	ftp://ftp.ncbi.nih.gov/gene/DATA/gene2go.gz
Ensembl IDs	NCBI Gene2ensembl	ftp://ftp.ncbi.nih.gov/gene/DATA/gene2ensembl.gz
Uniprot IDs	UniProt	ftp://ftp.uniprot.org/pub/databases/uniprot/current_release/knowledgebase/idmapping/by_organism/HUMAN_9606_idmapping.dat.gz
List of drugs binding to a protein	DrugBank	http://www.drugbank.ca/system/downloads/current/drugbank.txt.zip
List of pathways	REACTOME	http://www.reactome.org/download/current/uniprot_2_pathways.stid.txt
List of diseases	KEGG	http://www.genome.jp/kegg/
Pubmed articles citing a gene and their abstracts	pubmed2ensembl	http://www.pubmed2ensembl.org/
GO Term enrichment computation	BiNGO	http://www.psb.ugent.be/cbd/papers/BiNGO/Customize.html

### Individualized Page for Each PPI

A webpage of a PPI consists of two sections: an *automatically generated annotation section* with detailed annotations describing the interaction and its participant proteins, and a *wiki section* where users can discuss the interaction. The details of the annotation section from top to bottom are as follows (see Figure 1).

#### Biomedical Annotations

The top of the section gives a link to the PubMed record of the original publication reporting the interaction; this publication source is obtained from HPRD or BioGRID. Following that, the count of papers citing that publication is shown; this count is obtained from PubMed. The citation count is provided so as to give an idea of the extent of the scientific impact of that interaction. Sometimes the original publication is cited more for the experimental method than for the interactions itself, but this can be easily concluded by following the PubMed link to the original publication. Next, biologically and medically relevant characteristics of the two participant proteins are shown where available: PDB IDs and structure, Gene Ontology cellular component, molecular function and biological process terms at the GO Slim level, associated pathways from REACTOME, associated diseases from KEGG, and drugs binding to that protein from DrugBank [21]. These annotations provide useful information for analyzing the biological function of the given interaction. Additionally, links to corresponding pages of the genes in other databases, namely, Entrez gene [22], HPRD, Ensembl [23], and Uniprot, are provided.

#### GO Terms Enriched among Interacting Partners

**Figure 3:**
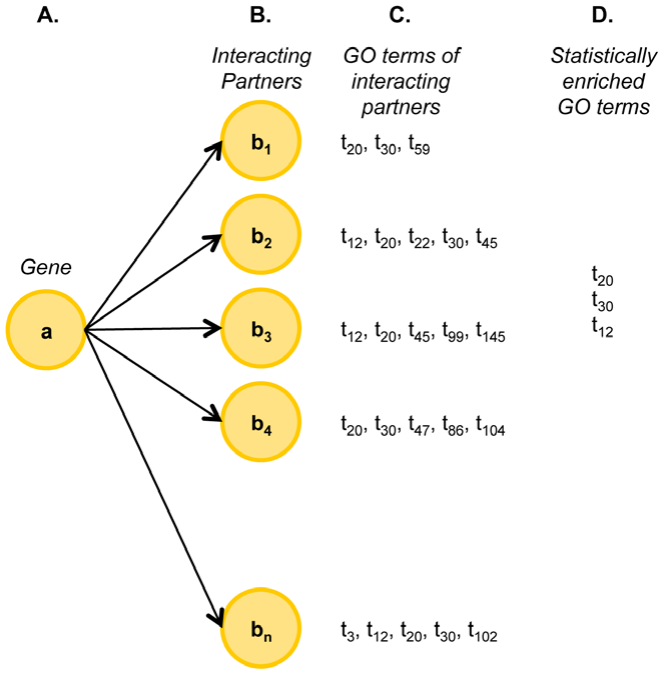
Concept diagram of GO term enrichment calculation. Gene *a* interacts with genes ,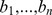-. GO terms t_i_ of each interacting partner are shown to its right. BiNGO computes the statistically enriched GO terms (functional categories that the genes are enriched in, and find that the statistically enriched GO terms are t_20_, t_30_, and t_12_.See methods in [24] for details of computation.

A unique feature of this web resource is that it provides for each gene in the interaction, a list of Gene Ontology biological process terms *statistically enriched* among its interacting partners. The enriched terms are computed by employing BiNGO plugin in Cytoscape [24], [25]. The hypergeometric statistical test of significance is used with a Benjamini & Hochberg False Discovery Rate (FDR) correction at a significance level of 0.05. For instance, when calculating enriched terms for gene ‘a’ (see Figure 3), the study group consists of the interacting partners b_1_, b_2_, …, b_n_, while the reference set consists of *n* genes randomly selected from the entire genome. BiNGO then collects GO biological process terms of b_1_, b_2_, …, b_n_. For each of the terms in the collection, it computes whether the number of genes associated with that term is significantly greater among interacting partners compared to that of random set. The methodology is described in detail in the original publication of BiNGO [24]. For a given gene (‘a’), if more than 50 terms are found to be enriched among interacting partners associations, only the top 50 enriched terms in the order of increasing p-value or decreasing statistical significance are shown on the website. For example, when viewing the annotations for an interaction between DISC1 and another protein, GO biological process terms that are significantly overrepresented in DISC1's interacting partners are shown. Viewing these terms would provide a handle for biologists in determining any novel associations of that gene in specific biological processes or diseases. These terms are especially useful when many interactions are known for a protein, but its functional characteristics are unknown [26].

#### Tag Clouds from Abstracts

To give an overview of the topics that each of these genes are associated with, *tag clouds* are presented which are constructed from abstracts of papers associated with each protein as given by pubmed2ensembl [26]. An interaction may be more interesting if it connects two different processes together, whereas it may be less novel if the interaction is between two proteins which participate in same biological process. Therefore, in addition to the above tag clouds, another tag cloud is displayed for each protein made up of words that associate with one protein but not the other. The tag cloud for a given gene is calculated as follows. First, a given gene's Ensembl identifier is mapped into a PMID (PubMed identifier) as given in pubmed2ensembl (http://www.pubmed2ensembl.org/) data. The abstract of the said publication is obtained, and it is treated as a document representing that particular gene. Starting with all of the abstracts as a corpus, stop words (such as ‘for’, ‘it’, ‘the’, etc) are removed, and stemming is carried out on the remaining words. Tf-idf, which is a measure of relevance used in information retrieval, is computed. *tf* refers to term-frequency and *idf* refers to inverse document-frequency, and tf-idf gives the relevance of a term to a given document ([27]). The size of a word in the tag cloud corresponds to the values of *tf-idf* for that term with respect to the document.

#### Wiki for Further Annotations by Users

The second section of the interaction page is the wiki, where users are encouraged to provide insights and discuss predictions about the relevance of the interaction in a biological process, disease or pathway. The wiki section may be used for crowdsourcing not only knowledge *curation* but also knowledge *creation* about each interaction.

## Navigation through Search

Users navigate Wiki-Pi primarily by using the search interface. Wiki-Pi allows full-text search as well as field-specific search; it does not require users to have the knowledge of any form of query language like Structured Query Language (SQL).

### 

#### Indexing for Information Retrieval

The index for free-text search is constructed from gene symbols, gene names, GO annotations, pathways, drugs, and diseases (but not enriched GO terms and abstracts). Stop words are removed and stemming is carried out on all the content prior to indexing. Stemming in the context of information retrieval is a process by which words like ‘inflammation’ and ‘inflammatory’ are mapped to their stem ‘inflamm’. When a word is queried, all interactions whose annotations (for either gene) contain that word are retrieved. The search functionality is created using the open-source search engine *Sphinx* (http://sphinxsearch.com/).

#### Search Functionality

**Figure 4:**
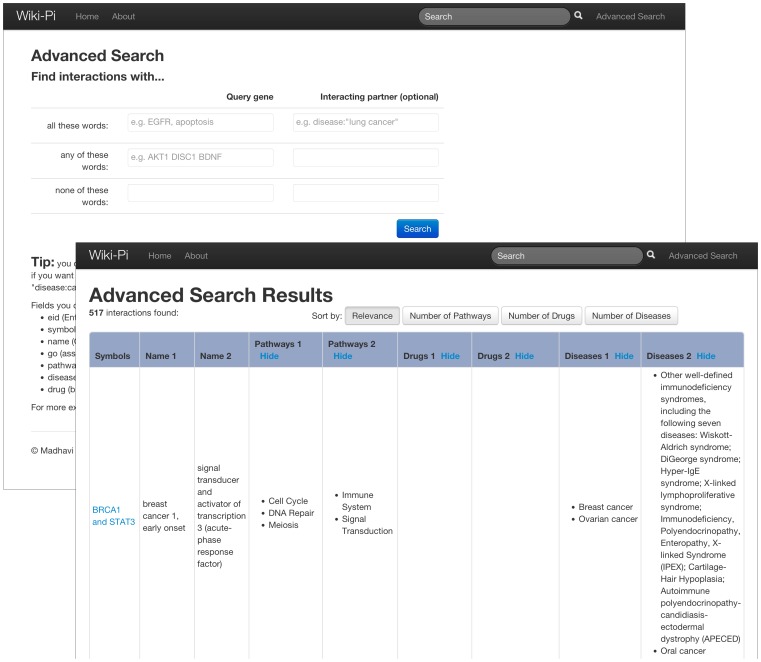
Advanced-search feature. Image shows the results of the search where one gene is involved in the immunity pathway, while the other gene contains the term cancer anywhere in its annotation but not the word immunity. Note that the results can be sorted by number of pathways, diseases or drugs associated with the genes (counts of each gene are considered individually). URL: http://severus.dbmi.pitt.edu/wiki-pi/index.php/search/adv?a-all=pathway%3Aimmunity&b-any=cancer&b-none=immunity.

The interactions may be retrieved with a simple search where any of the indexed content is given in the search box. For example, a query can be simply the gene symbol (e.g., AKT1) or any term that appears among the annotations of the gene (e.g. ‘blood’, ‘cytokine’, ‘hemostasis’). As stemming has been performed on all the words prior to indexing, searching for “inflammation” will retrieve interactions that have not only the word *inflammation* but also the word *inflammatory*. By allowing users to search for interactions based on fields such as GO terms, pathways, diseases, and drugs, researchers without a particular protein in mind can still successfully retrieve interactions of their interest. When multiple words are given in a simple search box, interactions containing all of the words are retrieved. An advanced-search page is also provided to retrieve interactions with more complex queries. Here, users can construct queries such as “DISC1 but not immunity”, “interactions of any of these proteins: TLR1, TLR2, …”, “genes associated with schizophrenia that interact with genes associated with immunity” and so on. An example is shown in Figure 4, where the query is: “an interaction where one gene is involved in the immunity pathway, while the other gene contains the term cancer anywhere in its annotation but not the word immunity”. Note that the users do not type such natural language sentences, but will type out query words in appropriate boxes in the advanced search page. Advanced search also allows users to restrict search to any of these fields: disease, pathway, drug, symbol, gene name, GO terms, or Entrez identifier (e.g. ‘disease:diabetes’, ‘pathway:hemostasis’ or ‘drug: diflunisal’).

#### Display of Search Results

**Figure 5:**
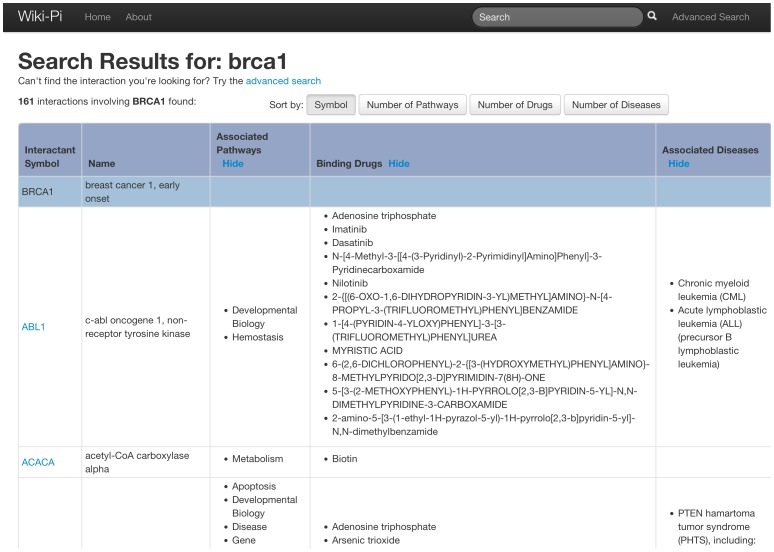
PPIs retrieved when searched by gene symbol. In these search results also, similar to those in Figure 3, the results can be sorted by number of pathways, diseases or drugs associated with the genes (counts of each gene are considered individually). URL: http://severus.dbmi.pitt.edu/wiki-pi/index.php/search?q=brca1.

**Figure 6:**
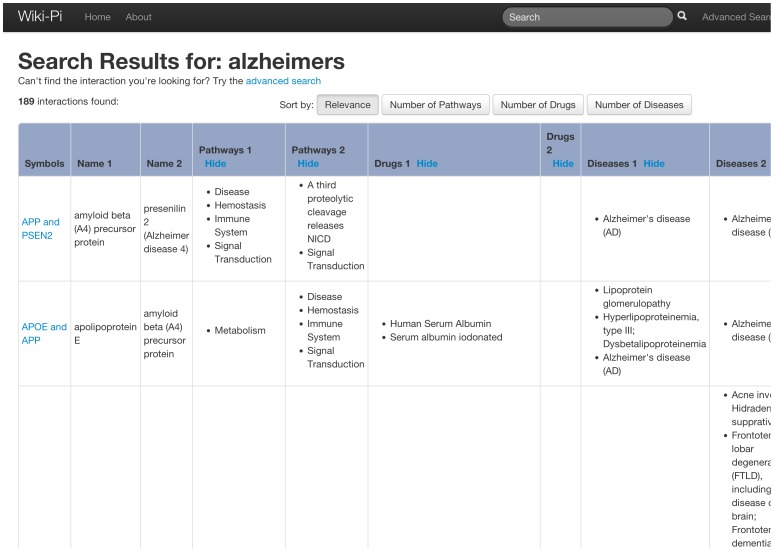
PPIs retrieved when searched by disease. In these search results also, similar to those in Figure 3, the results can be sorted by number of pathways, diseases or drugs associated with the genes (counts of each gene are considered individually). URL: http://severus.dbmi.pitt.edu/wiki-pi/index.php/search?q=alzheimers.

The results of the search are presented in a tabular format showing gene symbols, names, pathways, diseases and drugs of the participant genes (Figures 5 and 6). The rows are sortable by the number of attributes associated with the genes. Each interaction may be clicked to view the detailed annotations page of the interaction (Figure 1).

## Results and Discussion

### Formulation of Novel Hypotheses Uniquely Enabled by Wiki-Pi

Unique features available in Wiki-Pi enable addressing scientific queries that are otherwise not feasible by other tools. Without Wiki-Pi, a biomedical scientist is left with manual curation of information from several data sources without a guarantee on finding the seed evidence required to crystallize a novel hypothesis. A comparison of functionality in Wiki-Pi and those of other existing PPI databases is given in Table 2. Note that Wiki-Pi is the sole database that allows a user to search by specifying conditions about *both* the proteins involved in a given interaction. Imposing strict conditions on the interaction in effect narrows down the search space of PPIs; this is critical, as there are tens of thousands of PPIs available in existing databases. This capability is invaluable when hypothesizing functions of genes that are not well-studied.

**Table 2:** Comparison of functionality of Wiki-Pi with other PPI databases.

	SEARCHING FOR INTERACTIONS																	ANNOTATIONS ABOUT INTERACTIONS													
	Query on interaction characteristics					Query by characteristics of a gene						Query with constraints on characteristics of interacting partner						Results of a search			Details of each interaction										
	By protein name	By gene symbol	By experiment type	Grouping by family, of PPI partners	Multiple genes	Cellular Component/Subcellular Localization	Molecular Function	Biological Process	Associated Drugs	Associated Diseases	Associated Pathways	Cellular Component/Subcellular Localization	Molecular Function	Biological Process	Associated Drugs	Associated Diseases	Associated Pathways	Sorting Functionality	Data Export	Biomedical attributes shown for list of PPIs	Side-by-side annotations of both proteins	Experimental Evidence	Publication reporting the interaction	Impact achieved by that interaction (number of citations of original publication)	GO annotations of both proteins	Pathways of both proteins	Drugs binding to each of the two proteins	Diseases associated with both proteins	Tag-cloud from abstracts for both proteins	Cross references to external databases	Crowdsourcing further discussion about each PPI
**Wiki-Pi**	✓	✓			✓	✓	✓	✓	✓	✓	✓	✓	✓	✓	✓	✓	✓	✓		✓	✓	✓	✓	✓	✓	✓	✓	✓	✓	✓	✓
**STRING**	✓	✓		✓	✓																										
**IntACT**		✓	✓		✓	✓	✓	✓	✓										✓	✓		✓	✓								
**BioGRID**		✓																				✓	✓							✓	
**HPRD**	✓	✓				✓				✓								✓													

The search functionality and annotations displayed for the retrieved interactions are compared across different PPI databases. For each function, the cell shows a tick mark if the function is supported by the corresponding webserver.

Wiki-Pi is especially useful today, as several genome-wide association studies (GWAS) are being published. GWAS studies are unbiased by current scientific knowledge (i.e. they do not have literature-bias) and often implicate genes with currently unknown biological functions to be associated with the disease under study. The number of GWAS studies has increased rapidly in the past couple of years. So far, 1,309 publications have reported GWAS results on 674 traits or diseases (www.genome.gov/gwastudies [28], accessed 2012-July-17). Though extensive work is being carried out to identify the common genetic variants that influence various diseases or traits through GWAS, the role of these genes and the exact mechanism of their action are yet to be discovered. Very little information is available about some of the GWAS-identified genes in terms of their molecular function and biological process. Wiki-Pi enables researching each of these genes and provides novel insights that may not otherwise materialize except when a scientist knows all the multiple specialized domains involved.

#### Possible Function of IGSF21 and the Likely Mediators of Its Association to Diabetic Retinopathy

**Figure 7:**
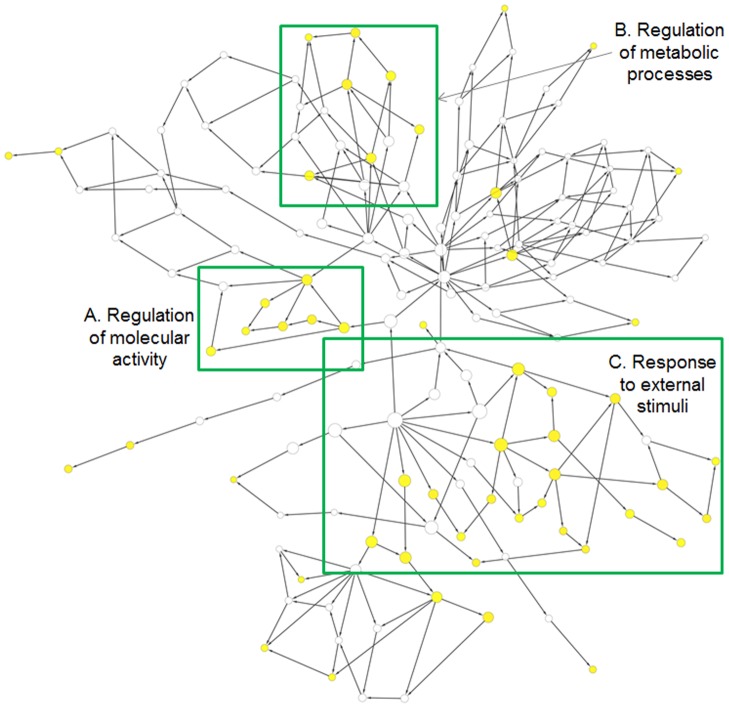
Statistically enriched Gene Ontology biological process terms of PPIs of IGSF21. Wiki-Pi website makes available only a list and not an image of enriched GO biological process terms. For clarification, this network diagram has been generated with BiNGO Cytoscape Plugin [24], for GO biological process terms, with the hypergeometric statistical test of significance, and a Benjamini & Hochberg False Discovery Rate (FDR) correction at a significance level of 0.05, by pasting the list of interacting partners (gene symbols) from Wiki-Pi. Statistical significance of the node (GO term) is shown in color, with the darker color indicating stronger significance. High-resolution image with labels of the nodes is available as File S1.

Using Wiki-Pi, we analyzed immunoglobin superfamily member 21 (IGSF21) which has been identified through a recent GWAS study to be associated with diabetic retinopathy, where new blood vessels form at the back of the eye causing bleeding and blurring of vision [29]. There is no information currently known about IGSF21 except for the protein-protein interactions determined through high-throughput experiments and that it is an extracellular protein. Searching on Wiki-Pi for interactions of IGSF21, and then viewing the list of GO terms enriched among its interacting partners reveals that this extracellular protein may be involved in regulating metabolic processes, catalytic activity as well as cytoskeletal organization and response to external stimuli (see Figure 7 and File S1, generated by pasting list of interacting partners of IGSF21 into Cytoscape BiNGO plugin [24]). Although this enriched term calculation reveals that IGSF21 may be involved in signaling mechanisms in response to external stimuli, specifically in cytoskeletal organization, it does not reveal its relation to diabetic retinopathy. Its relation specifically to diabetic retinopathy is revealed further with the advanced-search feature of Wiki-Pi, which may be used to find interactions where one gene is IGSF21 and the other gene includes the term “blood” in any of its annotations (http://severus.dbmi.pitt.edu/wiki-pi/index.php/search/adv?a-all=symbol%3Aigsf21&b-all=blood). This query results in four interactions, namely with (i) heat shock 27 kDa protein 1 (HSPB1), (ii) v-Ki-ras2 Kirsten rat sarcoma viral oncogene homolog (KRAS), (iii) thymosin beta 4 X-linked (TMSB4X), and (iv) diacylglycerol kinase delta 130 kDa (DGKD). The annotations of these four interacting partners on their corresponding interaction pages on Wiki-Pi show that HSPB1 is involved in *blood vessel endothelial cell migration* and the other three, namely KRAS, TMSB4X, and DGKD, are all involved in *blood coagulation*. Further, KRAS annotations show that it is involved in *insulin receptor signaling pathway* (GO biological process). Researching for these genes outside of Wiki-Pi (i.e. in PubMed), it is also found that (i) TMSB4X may play a role in diabetic retinal neovascularization in the context of proliferative diabetic retinopathy [30], and that (ii) DGKD deficiency causes peripheral insulin resistance and metabolic inflexibility [31]. We conclude that IGSF21 may be involved in signaling cellular response to external stimuli, specifically triggering cytoskeletal organization and regulation of metabolic and catalytic activity, and that its association to diabetic retinopathy may be mediated through its interactions with the genes HSPB1, KRAS, TMSB4X and DGKD which are involved in blood-coagulation.

### Conclusions

Wiki-Pi provides a means for effectively retrieving and studying human protein-protein interactions. The data itself is not curated by us, but is retrieved from other widely-used human protein information databases (Table 1). Wiki-Pi presents this information in a manner that is easy to be found and assimilated by biologists. The database is also timely because in the last few years several genome-wide association studies have been completed which resulted in the identification of genes associated with specific diseases or traits. Biological role of many of these genes is currently unknown or not fully characterized. If any such gene has known PPIs, the biological role of the gene may be determined based on the functions of its interacting partners.

Wiki-Pi facilitates the discovery of the molecular interconnects, if any, between seemingly unrelated biological processes that govern the human body: e.g. psychological stress and inflammation [32], [33], [34], [35], [36], [37], [38], or schizophrenia and immunity [32], [39], [40], [41]; although these processes are hypothesized to be related, the molecular pathways connecting these processes are not well understood. Wiki-Pi makes it possible to search for interactions connecting these processes.

Biologists routinely draw inferences by putting together the information about the proteins and formulate hypotheses and conduct experiments to validate them; Wiki-Pi makes assimilation of such information extremely easy by presenting all or most of the required annotations readily at hand. Wiki-Pi complements traditional databases, promoting research in molecular biology and biomedical informatics of human proteins. Future developments include the integration of additional data sources (both interactions and annotations) and the addition of authorship tracking for the wiki.

## Supporting Information

File S1 **Statistically enriched Gene Ontology biological process terms of PPIs of IGSF21.** This figure is generated similar to Figure 6, but the node labels are shown and the image is in high resolution. Statistical significance of the node (GO term) is shown in color, with the darker color indicating stronger significance.
(PDF)
