# Supplementary figures and images for: Wiki-Pi: A Web-Server of Annotated Human Protein-Protein Interactions to Aid in Discovery of Protein Function
Source: PLoS One. 2012 Nov 28;7(11):e49029. doi: 10.1371/journal.pone.0049029 (PMC3509123; doi:10.1371/journal.pone.0049029)

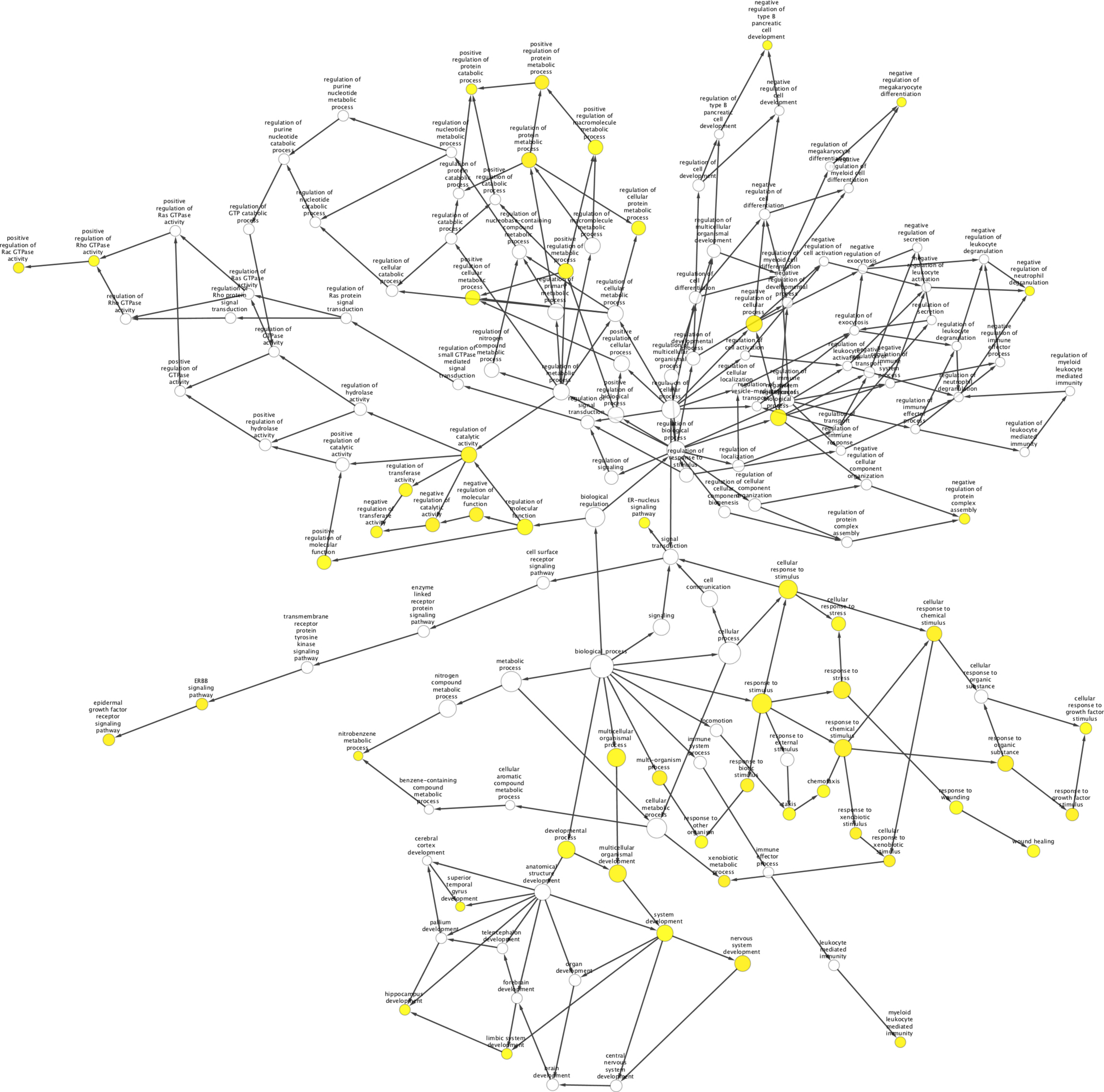

Supplement: File S1 — Statistically enriched Gene Ontology biological process terms of PPIs of IGSF21. This figure is generated similar to Figure 6, but the node labels are shown and the image is in high resolution. Statistical significance of the node (GO term) is shown in color, with the darker color indicating stronger significance. (PDF) [file pone.0049029.s001.pdf]
